# Comparison of Laser Pulse Duration for the Spatially Resolved Measurement of Coating Thickness with Laser-Induced Breakdown Spectroscopy

**DOI:** 10.3390/s19194133

**Published:** 2019-09-24

**Authors:** Carl Basler, Albrecht Brandenburg, Katarzyna Michalik, David Mory

**Affiliations:** 1 Fraunhofer Institute for Physical Measurement Techniques IPM, 79110 Freiburg, Germany; albrecht.brandenburg@ipm.fraunhofer.de; 2 LTB Lasertechnik Berlin GmbH, 12489 Berlin, Germany; katarzyna.michalik@ltb-berlin.de (K.M.); david.mory@ltb-berlin.de (D.M.)

**Keywords:** laser-induced breakdown spectroscopy (LIBS), coating thickness, pulse duration comparison, ultrashort laser pulses, industrial application

## Abstract

In this study, a method is presented to measure precisely the thickness of coated components based on laser-induced breakdown spectroscopy (LIBS). The thickness is determined by repetitively ablating the coating with ultrashort laser pulses, monitoring the spectrum of the generated plasma and calculating the coating thickness from the specific plasma signal in comparison to a reference measurement. We compare different pulse durations of the laser (290 fs, 10 ps, 6 ns) to extend the material analysis capabilities of LIBS to a real thickness measurement tool. The method is designed for production processes with known coating materials. Here, we show this for a nickel coating and a tungsten carbide coating on a copper sample with thicknesses from 5–30 µm.

## 1. Introduction

Laser-induced breakdown spectroscopy (LIBS) is an elemental analysis technique where a short laser pulse is focused onto the surface of a sample to transfer a small fraction of the surface in a plasma. Spectroscopic analysis of the plasma allows the identification of the contained elements. As the technique is all optical, it is fast, but still very sensitive. Compared to other elemental analysis techniques like X-ray fluorescence (XRF), glow discharge optical emission spectroscopy (GDOES), and secondary-ion mass spectrometry (SIMS), it has several advantages. GDOES and SIMS cannot be used inline and the speed is higher for LIBS compared to XRF. This is why LIBS is used in industrial applications such as metal sorting, alloy identification or purity control. In general, in these applications, a bulk material is analysed as an accumulation of plasma spectra generated by many laser pulses. However, LIBS is also capable of depth profiling [1,2,3,4,5] to analyse non-homogeneous material or coated material. Different attempts can be found in literature: with the variation of the intensity, the depth of the ablation crater varies and shows plasma signals from elements in different depths [6] (pp. 401–404). Another approach is the repetitive ablation of material with several pulses at the same location while monitoring the change in the plasma signal. This can be done either with nanosecond pulses [7,8,9,10], nanosecond double pulses [6,11] or femtosecond pulses [2,12,13]. While a nanosecond laser is generally used in LIBS setups, ultrashort pulse lasers (≤10 ps) create a much more defined ablation per pulse and show a better depth resolution [13]. In the literature, this is often referred to as cold ablation because within the short pulse duration the thermal diffusion length is determined by the diffusion of the electrons, and the energy is not yet transferred to the lattice [2]. This results in a smaller region of melt around the ablation crater and avoids a mixing of different layers of the sample. Disadvantages of ultrashort pulse lasers (USP laser) for LIBS are a lower available pulse energy and lower plasma temperature leading to a lower spectroscopic signal and probably X-ray radiation for fs laser pulses [14].

Here we show a setup with a USP laser developed to measure the thickness of coatings in the range of 1–100 µm for industrial applications. The measurements were done on different kinds of metal sheets with pulses between 290 fs and 6 ns. The plasma was analysed with a simple CMOS Czerny-Turner spectrometer. For each laser pulse (or burst of pulses) a plasma spectrum was recorded. As a measure of the coating thickness, we count the number of laser pulses needed for the lines of the base material to exceed a certain threshold. This signal is referenced to samples with a defined thickness, which were produced in a galvanization process. The reference measurement was done with a calibrated, commercially XRF device.

This technique can be used to control a coating process like galvanization or plasma coating with high depth and spatial resolution. For these coating processes, the thickness is not easily controlled and may become locally inhomogeneous. During galvanization, a locally inhomogeneous coating for the case of galvanization can occur due to the distribution of the field line density of the electric field. Standard XRF systems which are used in this field are not capable of resolving the local variations of the coating thickness. In the case of LIBS, the local resolution is as good as the size of the laser focus—in our case approximately 70 µm. With the rapidly rising market for USP lasers and similarly falling prices, this technology is also becoming increasingly interesting for industrial LIBS applications.

## 2. Materials and Methods

**Figure 1:**
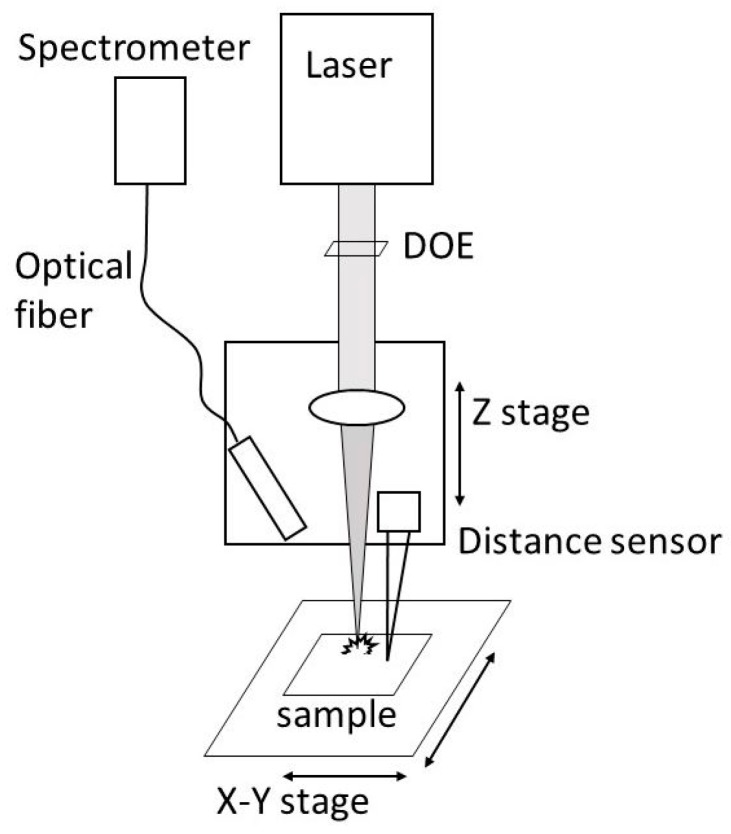
Experimental setup for laser-induced breakdown spectroscopy (LIBS).

A sketch of the experimental setup used for the coating measurement with LIBS is depicted in Figure 1. For the ablation of the material a commercial Yb:YAG laser system is used (Light Conversion, Carbide CB5–05, Vilnius, Lithuania). It delivers 85 µJ pulse energy and emits at the fundamental mode 1028 nm wavelength. The maximum repetition rate at full pulse power is 60 kHz. The pulse duration can be tuned from 290 fs to 10 ps. The power stability is <0.5% RMS, and the pulse to pulse stability is <0.5% RMS. To make a comparison between fs, ps, and ns pulses, a second laser beam composed of 6 ns laser pulses is employed for diagnosis of the coating thickness. A polarizing beam splitter cube is utilized to direct the second beam through the same focusing lens to the ablated area. This second laser is a Nd:YAG laser (Quantum Light Instruments, Q2-100, Vilnius, Lithuania) with 1064 nm wavelength, pulse to pulse stability of 0.5% RMS and tuned to 250 µJ pulse power to have low ablation. In this way both lasers beam power densities are approximately factor of 3 above the ablation threshold.

The beam of the laser is shaped to a rectangular top hat profile with a diffractive optical element (DOE) (Topag FBS2-50-1030, Darmstadt, Germany). With a top hat profile, ideally, material is only ablated from the bottom of the ablation crater, while a gaussian profile will always ablate material in each depth from the surface to the bottom of the crater. A focal length of 200 mm results in a spot size of 70 µm (FWHM). The beam profile adjustments are measured with a beam profiling camera. The actual distance to the sample was found by changing the distance between the focusing lens and sample and analysing the ablation profile under a 3D-laser scanning microscope. To keep the sample at the correct focal distance and to keep pulse energy density and ablation rate constant, a distance sensor monitors the distance. The signal directly controls the distance of the focusing lens of the ablation laser.

The sample can be moved with an X-Y stage to stitch an image of the coating thickness. The femtosecond laser is used in burst mode where the spectrum of a burst of pulses is analysed in order to speed up the measurement. This saves a lot of time for the measurement process as the readout time of the spectrometer limits the total speed. A Czerny Turner spectrometer with a CCD-Line (Avantes Avaspec ULS2048L-EVO, Avantes BV, Apeldoorn, The Netherlands) is used to detect the spectral information of the plasma. It has a 10 µm entrance slit and a spectral resolution of 0.1 nm. The used integration time is 1.05 ms, starts before the laser pulse and integrates over the whole plasma lifetime. The plasma imaging is done with a system containing two spherical lenses with a numerical aperture of 0.2, creating a 1:1 imaging from the plasma to the fibre.

Five copper samples were used to evaluate the method for coating measurements. The samples were galvanized with a 5–30 µm thick nickel coating and referenced to an XRF measurement [15] (pp. 554–600).

## 3. Results

### 3.1. Laboratory Implementation

The galvanized samples are rectangular, polished copper plates with 50 × 50 × 6 mm size. A thread on one side was used to connect it properly for the galvanization process (see Figure 8). Nickel coatings with thicknesses of 5, 10, 15, 25 and 30 µm were applied to the samples. The exact thickness was measured 4 times with a XRF device in the middle of each sample. The LIBS measurement was also done on 4 positions in the middle of the plate with a spacing of 500 µm between them. From the 4 measurements, a standard deviation for both techniques could be determined.

**Figure 2:**
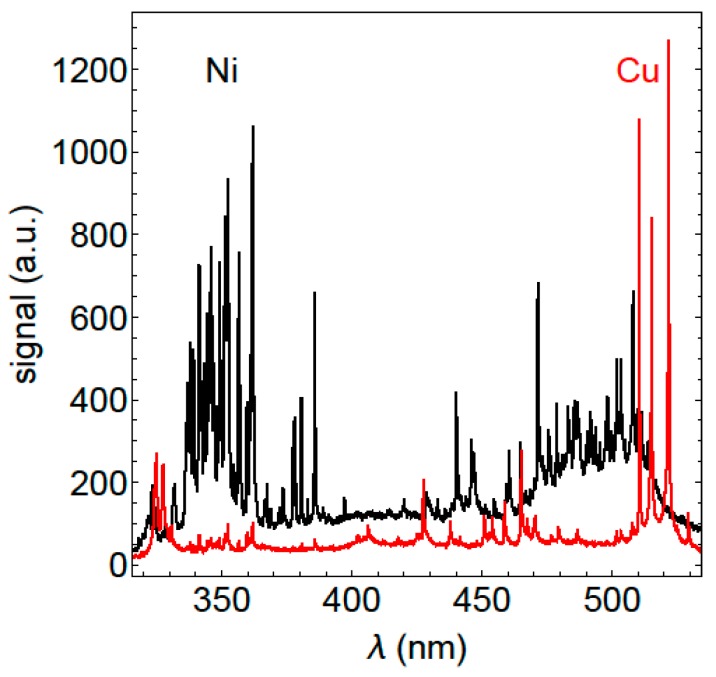
Spectrum of the plasma of a Ni coated copper sample, created with a burst of five 10 ps laser pulses. The black spectrum shows the second recorded spectrum, the red spectrum shows the 200th recorded spectrum.

The identification of the LIBS lines was done with spectra from samples of pure materials and its comparisons to lines in the NIST LIBS database [16]. For copper the lines 510.5, 515.3, 521.9 nm and for Ni the lines 339.3, 341.5, 344.6, 346, 349.3, 351.5, 352.4, 356.7, 361.9, 385.8, 471.4, 503.5 nm were used. The laser is used in burst mode, where 5 pulses are emitted with 60kHz and the spectrum of the plasma generated by these 5 pulses is accumulated on the detector. Figure 2 shows one spectrum of the second ablation burst (black spectrum, pure nickel spectrum) and one spectrum after 1000 pulses/200 bursts (red spectrum, pure copper spectrum).

**Figure 3:**
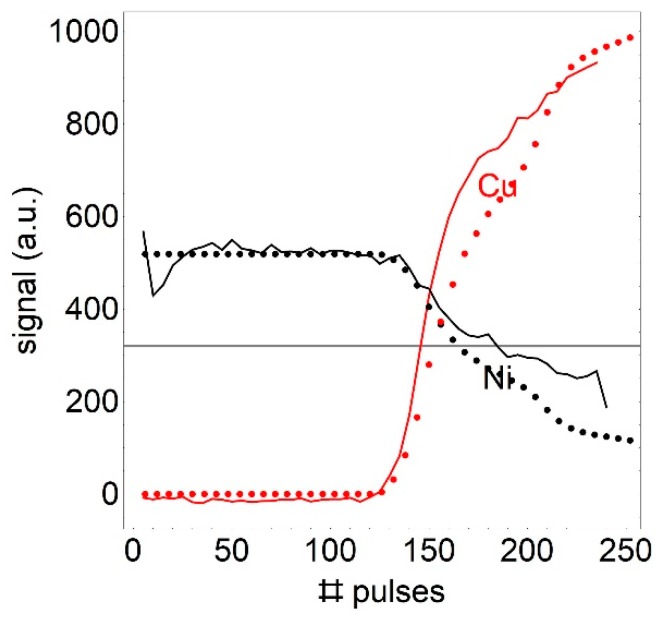
Average intensity of the selected Ni and Cu-lines as a function of the number of laser pulses for a 5 µm thick nickel coating, ablated with 10 ps pulses. Dotted lines are simulated based on the measured ablation profile.

In order to measure the layer thickness at one position of the sample, about 100 bursts of laser pulses are used to ablate the coating. Figure 3 shows the intensity at the position of the copper lines (red) and the nickel lines (black) as a function of laser pulses.

For the 5 µm thick nickel coating, the copper lines appear in the spectrum after 120 pulses with a rapid increase within the next 20 pulses. However, the decrease in the intensity of the nickel lines is much slower. The number of pulses needed to increase the signal of the copper lines above a certain threshold is chosen to be compared to the XRF reference value. This value is where the slope of the red line in Figure 3 is steepest and intensity fluctuations have the smallest effect. For the measurement with 290 fs this is 320 counts (Grey line in Figure 3), for 10 ps this is 500 counts and for 6 ns we chose 200 counts.

**Figure 4:**
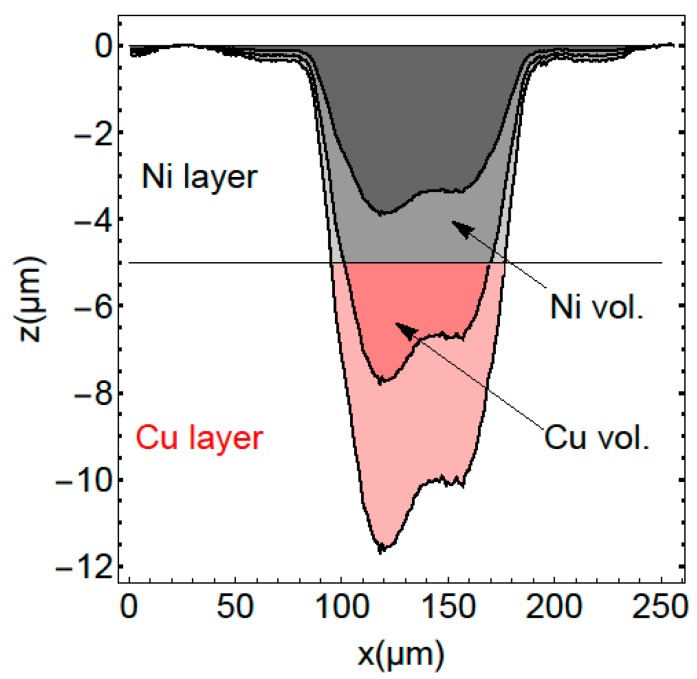
Laser crater cross-section mapped by a 3D laser-scanning microscope (upper profile). The lower profiles show the first profile scaled with factor of two and three respectively. The colours visualize the included area below (red)/above (grey) a certain threshold. The profile shows an asymmetry due to a slight misalignment of the top hat shaper.

In order to get some insight into the line intensity as a function of the ablation, we simulated the intensity based only on an experimental determination of the ablation profile. We assume that the measured ablation profile as shown in Figure 4 keeps its profile and just scales for processing ablation. In this figure, the three lines show a cross-section of the measured ablation profile. The upper line with dark grey filling shows the measurement, two scaled profiles are shown exemplary in Figure 4 with lighter grey and red filling. The filled colours mark the amount of coating and base material ablated in the corresponding pulse. The calculated volume corresponding to grey and red filled area in Figure 4 leads to a profile, which corresponds to the line intensity. The black dotted line in Figure 3 shows the simulated ablated volume per laser pulse of the upper coating material of a two- layer material based on the ablation profile of Figure 4. The red dotted line shows the amount of ablated material of the base material. The difference between simulation and measurement might be due to the fact, that we assume the same ablation rate for both materials.

**Figure 5:**
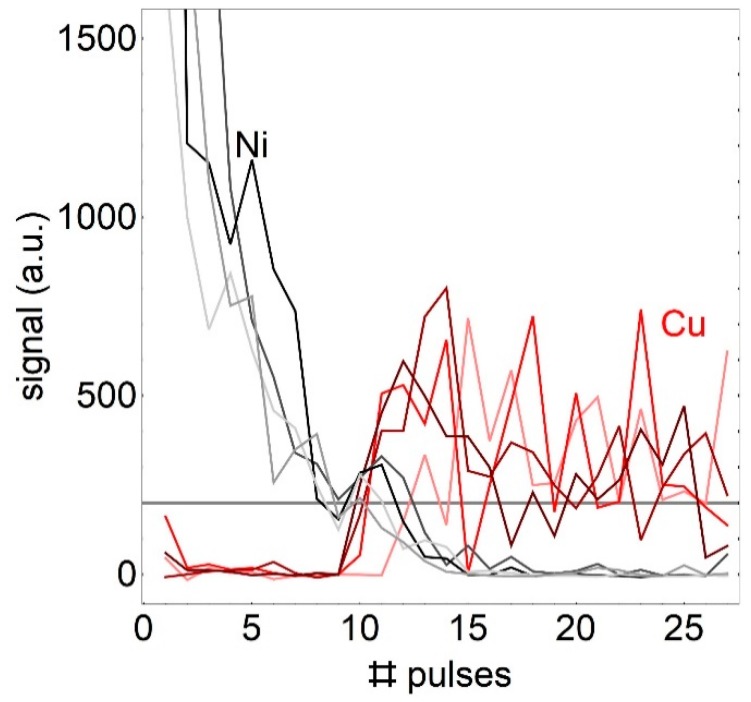
Intensity of Ni (grey tones) and Cu-lines (red tones) as a function of the laser pulses for a 25 µm thick nickel coating, ablated with 6 ns pulses. The 4 lines show a measurement at 4 different positions.

The line intensity as a function of the number of nanosecond laser pulses is depicted in Figure 5. The number of pulses is much lower than in the case of femto or picoseconds. The intensity variation is very high. We contribute this to a change in reflectivity and absorption of the laser pulse on the chaotic molten surface.

**Figure 6:**
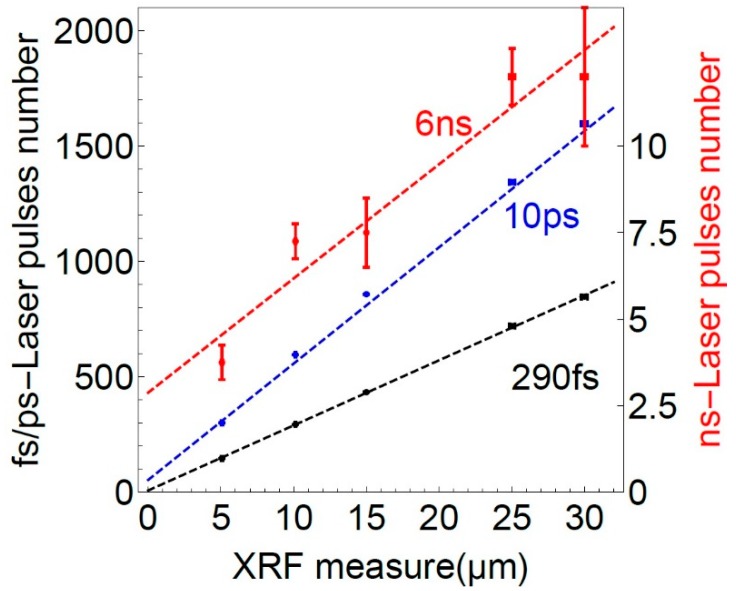
Number of pulses with LIBS as a function of the coating thickness. Black dots with 290 fs, blue dots with 10 ps, red dots with 6 ns. The standard deviation of the XRF measurement is about 0.1µm, the standard deviation for the 290 fs and 10 ps LIBS measurement is about 5 laser pulses and are thus not visible. Pulse number for ns-ablation on the right.

Figure 6 displays this number of laser pulses as a function of the thickness determined by an XRF measurement. The error bars show the standard deviation of the 4 performed measurements for every sample.

The variation of the LIBS measurement with 290 fs and 10 ps is typically about 1 recorded spectrum (one burst of 5 laser pulses) and thus error bars are not visible. The data for all pulse durations was fitted with the linear function:

(1) p(z)=a∗z+b,

The Table 1 shows the results of the fit. The parameter a is the slope in pulses per ablated micrometre, the parameter b describes the number of pulses needed to exceed the chosen signal threshold. The R2-value is good for 290 fs and for 10 ps, but shows a much lower reliability for the 6 ns pulses. The relative standard deviation of the fit parameters a and b is about one order of magnitude better for 290 fs compared to 10 ps, as the ablation depth seems to be not exactly linear with the number of pulses.

With the nanosecond laser the number of pulses does vary in a way which is not sufficient to distinguish between the samples. Femto- and picosecond laser show high reproducibility, however the accurate linear dependency seen with the femtosecond pulses is not present for the ablation with 10 ps pulses.

**Figure 7:**
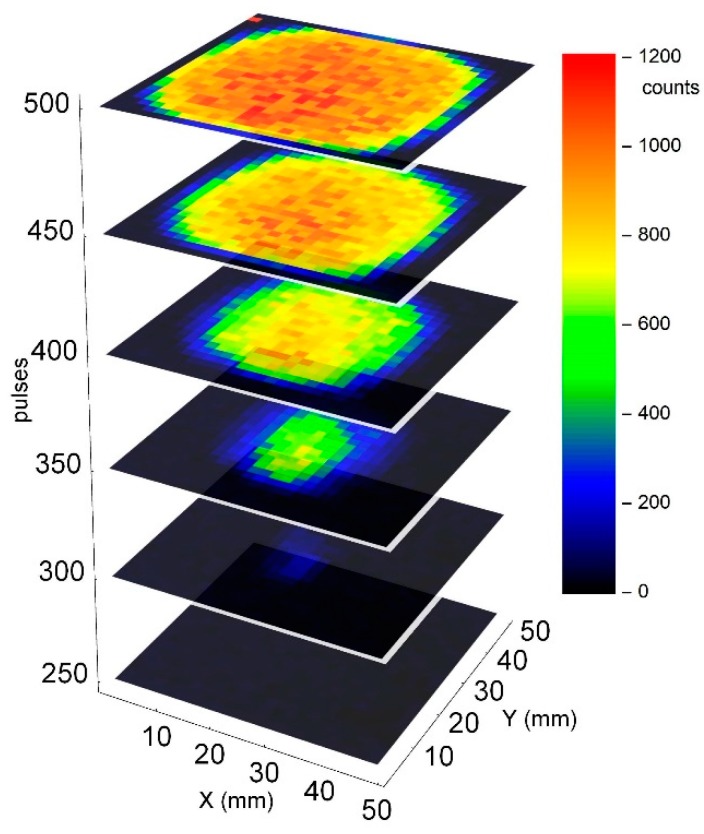
Mapped line intensity as a function of depth for a galvanized sample with 10 µm coating thickness in the centre.

Taking this data shown in Figure 6 to calibrate the system, we can now measure the coating thickness of the sample with high local resolution. Figure 7 shows the intensity of the copper lines as a function of the position on the sample and the number of the laser pulses at each position. 25 × 25 measurements are performed in steps of 1.85 mm across the surface.

**Figure 8:**
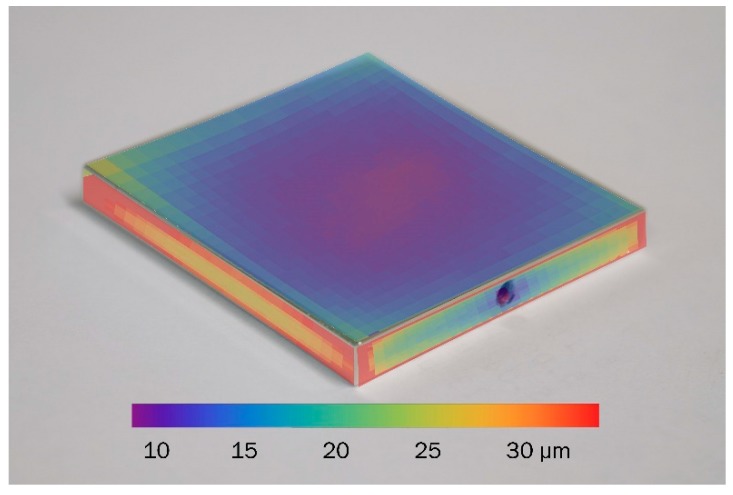
Resulting measurement of the coating thickness for a galvanized sample.

According to the calibration data from Figure 6 we can derive a thickness at all these positions. This is done in Figure 8, showing the thickness mapped onto a photograph of the sample.

### 3.2. Industrial Implementation

**Figure 9:**
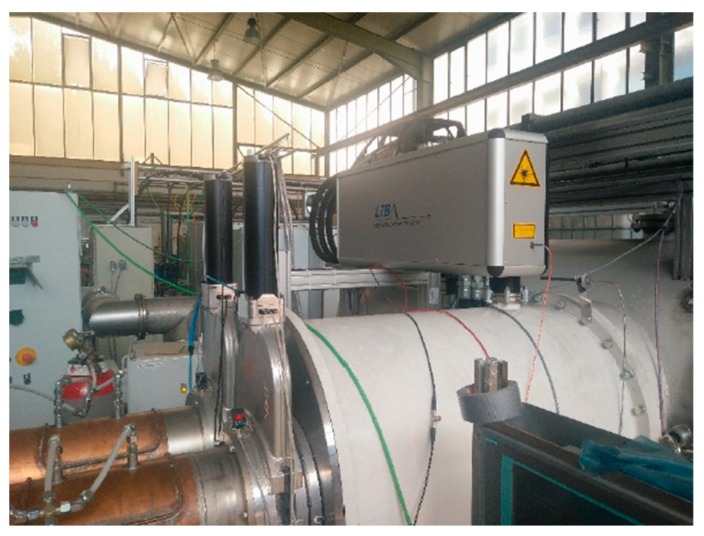
LIBS- system installed at plasma coating device.

The next phase of the project was to integrate the LIBS techniques and successful analysis methods into a system adapted to an industrial application. A prototype was designed and fabricated by LTB Lasertechnik Berlin and integrated into an existing vacuum chamber of a plasma coating system at Dr. Laure Plasmatechnologie GmBH (Figure 9).

Before testing, the industrial LIBS system was calibrated using copper coating samples of known thicknesses. During these tests, a Jobin Yvon spectrometer (CP140-1605, Horiba, Kyoto, Japan) with an ICCD camera (Andor iStar 734-18F03, Oxford Instruments, Abingdon, UK) was used. The used laser pulse duration was set to 290 fs.

**Figure 10:**
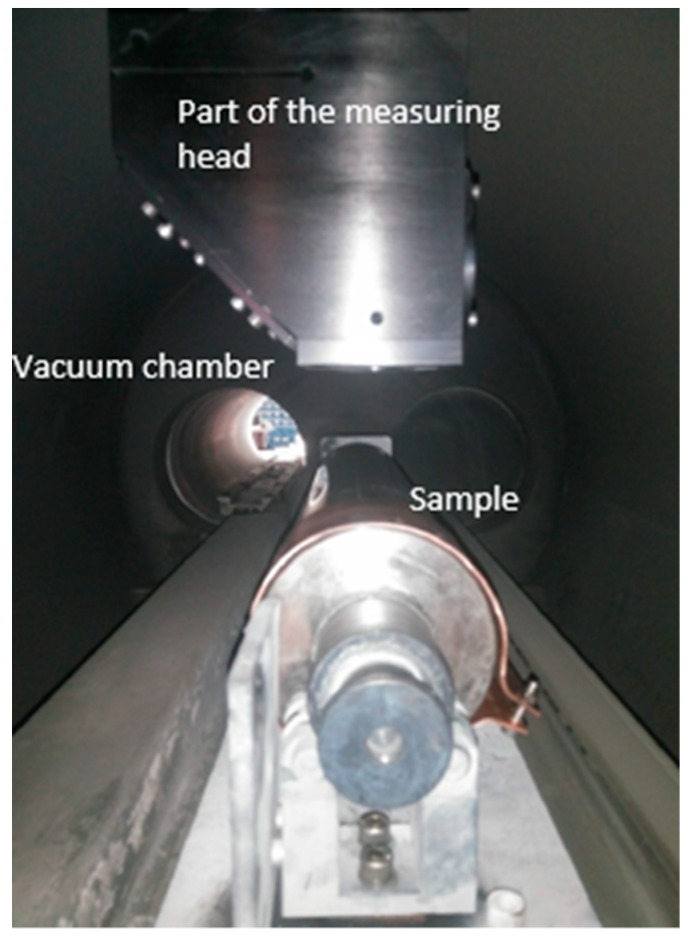
Copper ring sample in the vacuum chamber before coating.

**Figure 11:**
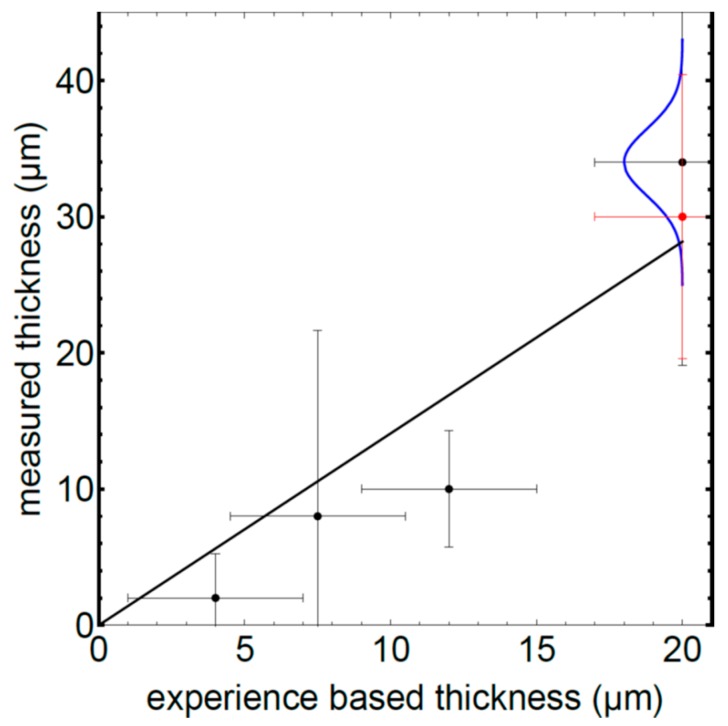
Results of tests. Black points: measured coating thickness with LIBS as a function of the appointed thickness. The x-values are experience-based values for the used parameters of the plasma chamber. The typical error is 3 µm. The y-values show the median of the measurements with the LIBS system. The red dot shows a reference measurement with a micrometer screw gauge. The blue curve shows the result of a 3d surface map measured with a laser scanning microscope.

Figure 10 shows the Copper ring sample, which was used to validate the system in the industrial setup. The sample was coated with tungsten carbide four times. After each coating, the thickness has been measured with LIBS, without opening the vacuum chamber. A series of five measurements was carried out. For the first three coatings, the medians of each of the measurements are within the required tolerance range. It was observed, however, that any inhomogeneity in the coating leads to strong variation of the LIBS measurements on a single sample. Nevertheless, a meaningful result can be achieved by averaging five measurements. Figure 11 shows the results of the LIBS measurement (black dots). The error bars on the y axes show the standard deviation for several measurements at different positions. This variation is confirmed by a reference measurement with a micrometer screw gauge (red dots) with similar variation at different positions. In addition, we have recorded a 3d microscope image of 300 × 400 µm and evaluated the surface roughness averaged over an area of the spot size of the LIBS laser. This leads to an expected height distribution shown with the blue curve in Figure 11. The black line is a fitted straight line though the origin to guide the eye.

## 4. Discussion and Conclusions

We demonstrated that sub-picosecond ablation is the preferred ablation pulse duration for the measurement of the coating thicknesses with LIBS. This is confirmed by an XRF measurement as a reference. With USP-Lasers LIBS is a useful tool for the spatially resolved measurement of coating thicknesses. Using femtosecond pulses the number of pulses needed to ablate the coating depends linearly on the thickness of the sample. Thus a single calibration measurement for a given material combination is sufficient to use this method for a wide range of coating thicknesses. For this pulse duration the curve of the LIBS signal is well understood and corresponds to the ablation profile. For 10 ps laser pulses the method gets slightly more inaccurate and does not work well for 6 ns pulses.

The material combination and the thickness range was chosen because the XRF works well for these samples. However, we applied this method also for samples in a range between 1 and 100 µm. For the determination of a coating thickness in this range, LIBS is a very fast and accurate method that can be implemented in an industrial environment. The measured variation in the industrial application is plausible and seems to show the local variation of the coating.

## Figures and Tables

**Table 1:** Fit parameters for the data set shown in Figure 6.

Pulse Duration	a (Pulse num./µm)	b (Bulse num.)	R^2^
290 fs	28.2 (±0.3)	7.8 (±5.5)	0.96
10 ps	50.4 (±3.6)	53.6 (±70.8)	0.95
6 ns	0.33 (±0.05)	2.88 (±0.96)	0.86
